# A simple and reliable protocol for long-term culture of murine bone marrow stromal (mesenchymal) stem cells that retained their in vitro and in vivo stemness in long-term culture

**DOI:** 10.1186/s12575-019-0091-3

**Published:** 2019-02-01

**Authors:** Basem M. Abdallah, Abdullah M. Alzahrani, Ashraf M. Abdel-Moneim, Nicholas Ditzel, Moustapha Kassem

**Affiliations:** 1 Biological Sciences Department, College of Science, King Faisal University, Hofuf, Al-Ahsa 31982 Saudi Arabia; 2 Endocrine Research (KMEB), Department of Endocrinology, Odense University Hospital and University of Southern Denmark, Odense, Denmark; 3 Department of Zoology, Faculty of Science, Alexandria University, Alexandria, Egypt; 4 Department of Cellular and Molecular Medicine, DanStem (Danish Stem Cell Center), Panum Institute, University of Copenhagen, Copenhagen, Denmark; 5 Stem Cell Unit, Department of Anatomy, Faculty of Medicine, King Saud University, Riyadh, Saudi Arabia

**Keywords:** Stem cells, BMSC, Osteoblast, bFGF, Ectopic bone

## Abstract

**Background:**

Bone marrow derived stromal stem cells (BMSCs) are a clonogenic cell population that is characterized by self-renewal capacity and differentiation potential into osteoblasts, and other mesenchymal cell types. Mouse BMSCs (mBMSCs) are difficult to be cultured and propagated in vitro due to their replicative senescent phenotype, heterogeneity and high contamination with plastic adherent hematopoietic progenitors (HPCs). In this study, we described long-term culture of homogenous population of mBMSCs using simple and highly reproducible approach based on frequent subculturing (FS) at fixed split ratio in the presence of basic fibroblast growth factor (bFGF).

**Results:**

Cultured mBMSCs using this protocol (mBMSCs-FS) showed long-term survival in culture > 70 population doubling (PD) and retained their characteristic surface markers and differentiation capacity into osteoblast and adipocyte lineages. When compared to the clonal bone marrow-derived cell line ST2, mBMSCs-FS displayed more enhanced osteoblast differentiation potential and responsiveness to osteogenic factors including BMPs, IGF-1, PDGF, TGFβ1,3, FGF, cAMP, Wnt3a and VEGF. In addition, unlike ST2 cells, mBMSCs-FS maintained capacity to form ectopic bone and bone marrow stroma upon in vivo transplantation in immune-compromising mice, even at high PD levels. Interestingly, by applying the same FS + bFGF protocol, we succeeded to obtain long-term cultures of primary neonatal calvarial osteoprogenitor cells (OBs) that were cultured for more than 70 PD and maintained in vitro and in vivo osteoblast differentiation capacities.

**Conclusions:**

Our data provide a simple and reliable protocol for generating long-term cultures of mBMSCs and OBs with retained high in vitro and in vivo osteoblast differentiation capacities for use in pre-clinical and molecular mechanism studies.

**Electronic supplementary material:**

The online version of this article (10.1186/s12575-019-0091-3) contains supplementary material, which is available to authorized users.

## Background

Adult stromal stem cells isolated from bone marrow (also known as skeletal stem cells, BMSCs) are a group of low frequent cells that have high capacity of differentiation into various mesoderm-cell types including osteoblast, adipocyte and chondrocyte cell lineages. BMSCs have been successfully used in regenerative medicine and tissue engineering, due to their differentiation capacity, immune-modulatory functions and paracrine effect [1, 2].

Isolation and culturing of mouse-derived BMSCs (mBMSCs) are crucial steps for investigating the mechanisms controlling the molecular cell determination of osteogenesis using bone-related transgenic mouse models. However, factors including the low frequencies of BMSCs in bone marrow, contamination with plastic adherent hematopoietic progenitors (HPCs) and replicative senescence phenotype hampered the culturing of large amount of mBMSCs in pure yield that can be used efficiently in different molecular and cellular studies [3, 4].

Several techniques have been used to isolate BMSCs from mouse bone marrow, including using different plating density [5], negative and positive selection based on specific surface marker expression [6–8], frequent medium change in primary culture [9], passage-dependent reseeding following trypsinization [10], centrifugation on a Percoll gradient [11] and cell sorting for CD29 (Itgb1) and CD54 (Icam1) [12]. However, these methods are not simple, need special requirements and do not always result in long-term cultures of BMSCs.

On the other hand, several immortalized cell lines with the characteristics phenotype of mBMSCs including in vitro multi-lineage differentiation potential have been established from mouse bone marrow. These clonal cell lines include for example, ST2 cells [13], mesenchymal progenitor cell line C3H10T1/2 [14] and TBR31–2 cell [15]. However, immortalized status of these cell lines significantly affected the maintenance of BMSCs physiological function in vivo. For example, the ST2 cell line has been extensively used as a model of (mBMSCs) in vitro, despite lacking the capacity for in vivo ectopic bone formation, except in the presence of BMP2 upon transplantation [16]. Thus, there is a need for developing simple and fast protocol for culturing and expanding large number of mBMSCs in long term with intact in vitro and in vivo multi-lineage differentiation.

In this study, we described simple and reproducible protocol for long-term culture of BMSCs/osteoprogenitors based on FS in the presence of bFGF. Using this protocol, we obtained long-term culture of mBMSCs and mOBs with retained high osteogenic differentiation capacity in vitro and in vivo at high passage number.

## Results

### Simple protocol for culturing and expanding murine BMSCs in long term

**Fig. 1:**
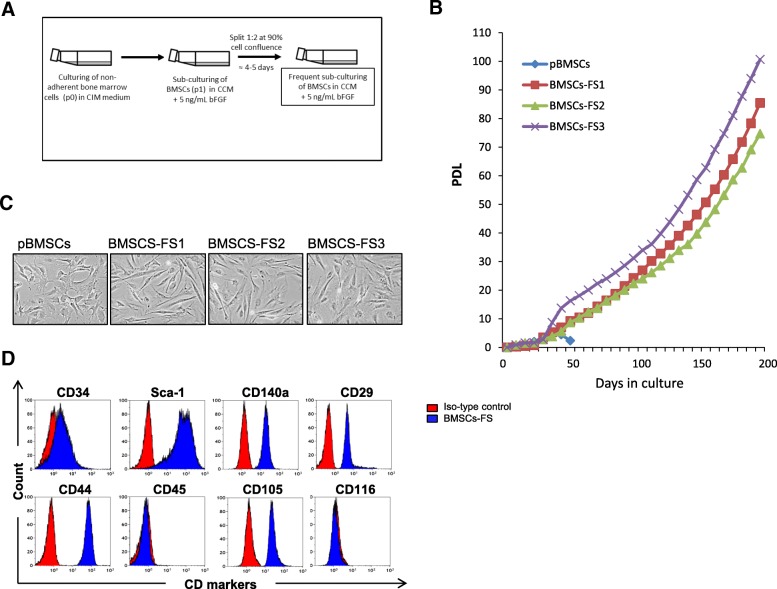
Long term culturing of primary mBMSCs using a combination of FS and bFGF. **a** Workflow of simple and reliable protocol used to culture and expand primary mBMSCs in long term. **b** Long term growth curves of primary isolated mBMSCs cultured by traditional method and three independent BMSCs-FS cell lines cultured by our new protocol in the presence of bFGF. **c** Phase contrast representative images (20x) of pBMSCs (p5) and three independent BMSCs-FS cell lines (at 70 PDL, p25). **d** FACS analysis of the expression of MSC surface markers by BMSCs-FS, p25. Values are mean ± SD of three independent experiments

We aimed to develop a simple, fast and highly reproducible protocol for culturing and expanding large number of mBMSCs for mechanistic and preclinical translational studies. We culture the primary mBMSCs using the traditional method of plastic adherence after avoiding the fast-adhered HPCs [17]. Cells were then, frequently sub-cultured at split ratio 1:2, when reached 90% confluence in 5 ng/mL bFGF for long term (Fig. 1a). We have applied this protocol to culture and expand mBMSCs derived from 3 independent mice. As shown in Fig. 1b, long-term growth curves of the three different cultures of mBMSCs (mBMSCs-FS1 to 3) showed long-term culture for more than 80 PDL during a period of approximately 150 days without growth arrest. On the other hand, culture of primary mBMSCs (pBMSCs) using traditional method without bFGF experienced senescent association growth arrest after 6 PDL (Fig. 1b). BMSCs-FS at p25 (70 PD) displayed homogenous population with spindle shape like structure as compared to the heterogeneous, large and flat shaped primary cell population of pBMSCs (p5) (cultured without FGF) as assessed by phase contrast microscopic analysis (Fig. 1c). Furthermore, BMSCs-FS at p25 expressed the characteristic surface markers of mouse mesenchymal stem cells including Sca-1, CD140a, CD29, CD44, and CD105 and did not express hematopoietic marker CD45 as measured by FACS analysis (Fig. 1d).

### Increased in vitro osteogenic potential of BMSCs-FS at high PDL

**Fig. 2:**
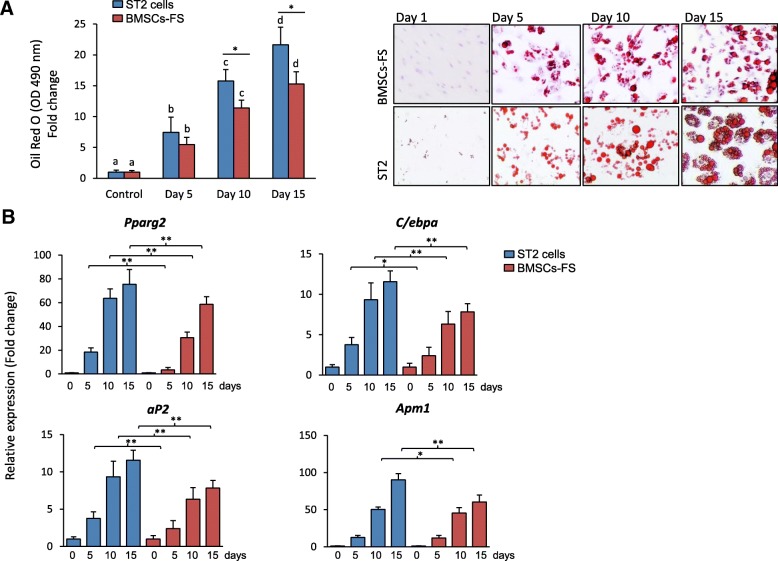
Long term cultured BMSCs-FS differentiates efficiently into adipocytic cell lineage. **a** Adipocyte differentiation of BMSCs-FS (p25) as compared with ST2 cell line. Cells were induced with adipogenic cocktail (M&M) and accumulation of fat droplets during the time course of differentiation were measured by quantitative Oil Red O staining. Representative images of Oil Red O staining are shown at each time point during adipogenesis of BMSCs-FS and ST2 cells. **b** qPCR analysis of the adipogenic markers expression in BMSCs-FS and ST2 cells after 12 days of adipogenic induction. Data were represented as fold change over control non-induced. Values are mean ± SD of three independent experiments, (*p < 0.05, compared to ST2 cells at the same time points). Columns of the same group with different letters at each time point indicate significant differences according to Duncan’s multiple range tests at *p* < 0.05

**Fig. 3:**
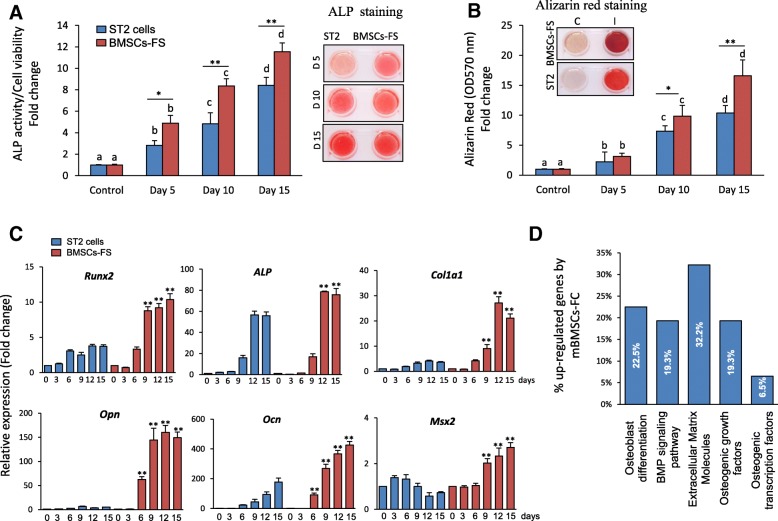
Increased osteogenic differentiation capacity in BMSCs-FS as compared with ST2 cells. **a** Osteoblast differentiation of BMSCs-FS (p25) as compared with ST2 cell line. Cells were induced with osteogenic cocktail and osteogenesis was measured by quantitative alkaline phosphatase activity (ALP) and (**b**) Alizarin red staining. ALP and Alizarin red measurements were normalized to the cell number. ALP and Alizarin red staining images are shown. **c** qPCR analysis of the osteogenic markers mRNA expression after 12 days of induction in BMSCs-FS compared to ST2 cell line. Each target gene was normalized to reference genes and presented as fold change over control induced. Date were represented as fold change over control non-induced. **d** Up-regulated osteogenic genes in BMSCs-FS versus ST2 cells after 6 days of osteogenic induction. Gene expression was measured by qPCR using osteogenic RT2 profiler array as described in M&M. Values are mean ± SD of three independent experiments, (*p < 0.05, **p < 0.005, compared to ST2 cells at the same time points). Columns of the same group with different letters at each time point indicate significant differences according to Duncan’s multiple range tests at p < 0.05

To study whether BMSCs-FS can maintain their multilineage differentiation capacities at high PDL, we compared the osteogenic and adipogenic differentiation of BMSCs-FS (at PDL 70, p25) with the commercially available immortalized ST2 cell line. As shown in Fig. 2a, both cell lines could differentiate efficiently into mature adipocytes as assessed by increased Oil red O staining for the accumulation of lipid droplets and significant up-regulation of early (*Pparγ2* and *C/ebpα*) and late (*aP2, Apm1*) adipogenic gene expression markers (Fig. 2 a & b). However, the differentiation capacity of BMSCs-FS (p25) into adipocytes was less than that of ST2 cell line as shown by reduced the accumulation of fatty acids and expression of adipocytic markers (Fig. 2 a & b). In contrast, BMSCs-FS (p25) showed high osteogenic differentiation potential compared to ST2 cell line, as evaluated by significant increased levels of ALP activity and formation of matrix mineralization (Fig. 3a & b). Quantitative mRNA expression levels of early (*Runx2, Msx2, Dlx5*, and *Alp*) and late osteoblastic markers (*Ocn* and *Opn*) were significantly up-regulated in BMSCs-FS compared to ST2 cells (Fig. 3c). In consistent, qPCR-based osteogenic gene array analysis revealed the upregulation of 67.5% (≥2 fold, *p* < 0.05) of the differentially expressed osteoblastic genes in BMSCs-FS during their osteogenesis compared to ST2 cells (Fig. 3d, Table 1 and Additional file 1: Table S2). The up-regulated genes by BMSCs-FS included osteogenic growth factors, osteoblast differentiation and BMP signaling related-genes that represent 19.3, 22.5, and 19.3% respectively of total upregulated genes (Fig. 3d).

**Table 1:** Upregulated osteoblastic genes by BMSCs-FS (p25) versus ST2 cells

Gene name	Gene symbol	Fold
Ossification and matrix molecules		
Alkaline phosphatase, liver/bone/kidney	*Alpl*	7.9
Bone gamma carboxyglutamate protein	*Bglap*	2.1
Collagen, type I, alpha 1	*Col1a1*	7.5
Collagen, type I, alpha 2	*Col1a2*	4.2
Fibroblast growth factor receptor 1	*Fgfr1*	4.4
Fibroblast growth factor receptor 2	*Fgfr2*	2.3
Insulin-like growth factor I receptor	*Igf1r*	2.2
BMP signaling pathway		
Bone morphogenetic protein 2	*Bmp2*	74
Bone morphogenetic protein 3	*Bmp3*	25.3
Bone morphogenetic protein 5	*Bmp5*	2
Bone morphogenetic protein 6	*Bmp6*	17.7
Bone morphogenetic protein receptor, type 1B	*Bmpr1b*	3.8
Bone morphogenetic protein 4	*Bmp4*	2
Extracellular Matrix (ECM) Molecules		
Biglycan	*Bgn*	2
Collagen, type II, alpha 1	*Col2a1*	7.5
Collagen, type III, alpha 1	*Col3a1*	2.2
Collagen, type XIV, alpha 1	*Col14a1*	2.2
Collagen, type V, alpha 1	*Col5a1*	2.6
Integrin alpha 2	*Itga2*	10
Integrin alpha 2b	*Itga2b*	1.5
Matrix metallopeptidase 2	*Mmp2*	17
Matrix metallopeptidase 8	*Mmp8*	12
Matrix metallopeptidase 9	*Mmp9*	23.7
Osteogenic growth factors		
Colony stimulating factor 1 (macrophage)	*Csf1*	3
Fibroblast growth factor 1	*Fgf1*	2.5
Fibroblast growth factor 2	*Fgf2*	2.7
Growth differentiation factor 10	*Gdf10*	3.7
Insulin-like growth factor 1	*Igf1*	2
Platelet derived growth factor, alpha	*Pdgfa*	3.2
Osteogenic transcription factors		
Runt related transcription factor 2	*Runx2*	4.5
Sp7 transcription factor 7	*Sp7*	6.7

Cells were induced into osteoblast differentiation as described in the M&M. Mouse osteogenesis RT^2^ Profiler™ PCR array with 84 osteogenic genes was performed using the SYBR® Green quantitative PCR method. Each target gene was normalized to a group of reference genes as described by manufacture instruction’s. Up-regulated genes (≥ 2 fold) by BMSCs-FS are represented as fold change over ST2 cells

**Fig. 4:**
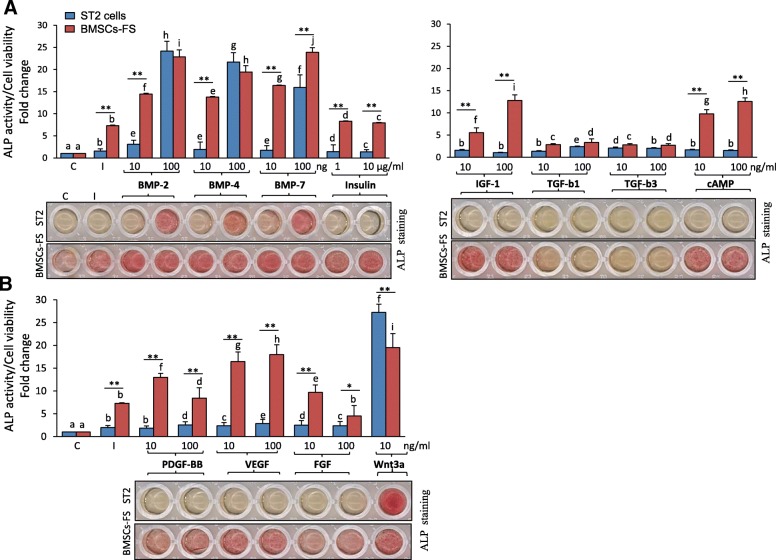
Increased the response of BMSCs-FS to osteogenic signaling factors. **a**, **b** Response of BMSCs-FS (p25) Vs ST2 cells to different osteogenic growth factors as measured by quantitative ALP activity. Cells were either cultured without osteogenic induction media (C, control), or induced to osteogenic lineage in the absence (I, induced) or the presence of 10 and 100 ng/mL of different osteogenic growth factors for 6 days. Representative images of ALP staining are shown. ALP measurements were normalized to cell number and represented as fold change over control non-induced cells. Data are mean ± SD of three independent experiments. (*p < 0.05, **p < 0.005, compared to ST2 cells with the same condition). Columns of the same group with different letters at each growth factor indicate significant differences according to Duncan’s multiple range tests at *p* < 0.05

We further compared the osteogenic response of BMSCs-FS (p25) and ST2 toward a variety of known osteogenic signaling molecules. As shown in Fig. 4a & b, BMSCs-FS treated with BMP-7, Insulin, IGF-1, cAMP, PDGF-BB, VEGF and FGF at both 10 and 100 ng/mL (and 1 and 10 μg/mL for insulin) showed significantly increased ALP activity compared to ST2 cells (Fig. 4a & b). In addition, BMSCs-FS showed significant increased ALP activity in response to both BMP-2 and BMP-4 at 10 ng/mL as compared to ST2 cells (Fig. 4a). On the other hand, the effects of both TGF-β1 and TGF-β3 at 10 and 100 ng/mL and both BMP-2 and BMP-4 at 100 ng/mL on ALP activity were comparable between BMSCs-FS and ST2 cells (Fig. 4a). ST2 cells showed significant high ALP activity as compared to BMSCs-FS in response to Wnt3a (Fig. 4b).

### BMSCs-FS retains their stemness in vivo at high PDL compared to ST2 cell line

**Fig. 5:**
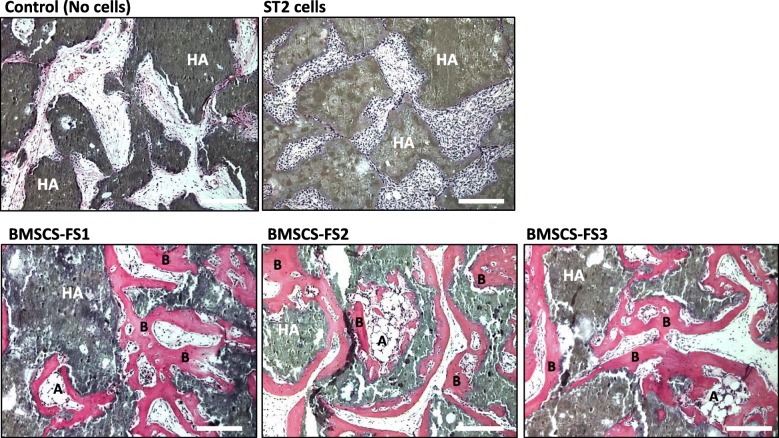
Maintained in vivo stemness of BMSCs-FS at high PDL. In vivo ectopic bone formation by implanted BMSCs-FS (70 PDL, p25) and ST2 cells. Cells were mixed with HA/TCP powder and implanted subcutaneously in immune-deficient mice for 2 months (*n* = 6 implants for each cell line). Histological sections of implants were stained with H&E. Representative images of H&E sections show the in vivo capacity of 3 independent BMSCs-FS to form in vivo ectopic bone and bone marrow stroma containing adipocytes and fibroblasts. B = Bone, A = Adipocyte, HA = Hydroxyapatite. Scale bars indicate 100 μm

The capacity of BMSCs to form ectopic bone and bone marrow stroma in vivo, is a standard assay for verifying the stemness of skeletal stem cells [18]. Thus, we mixed ST2 cells and BMSCs-FS (three different cell lines at PDL ≥ 70, p25); with HA/TCP powder and implanted them subcutaneously in immune-deficient mice for two months. As shown in Fig. 5a, histological analysis of BMSCs-FS implants revealed the formation of heterotopic bone and bone marrow stroma including adipocytes. On the other hand, ST2 cells were unable to differentiate into bone in vivo (Fig. 5).

### Long-term culture of murine osteoprogenitors using FS and bFGF protocol

**Fig. 6:**
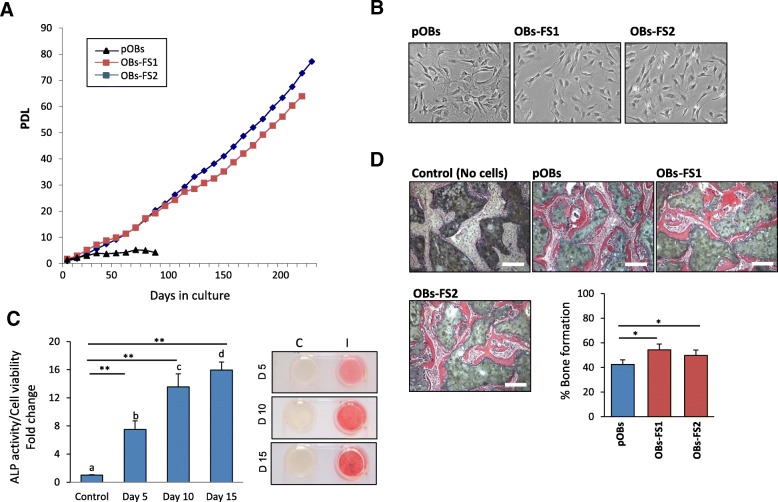
Long term culturing of primary neonatal calvarial osteoblasts (OBs) using a combination of frequent subculture and bFGF. **a** Long term growth curves of primary isolated OBs cultured by traditional method and two independent OBs-FS cell lines cultured by our new protocol in the presence of bFGF. **b** Phase contrast representative images (20x) of OBs (p5) and two independent OBs-FS cell lines (at 50 PDL, p30). **c** Osteoblast differentiation of OBs-FS, p30 measured by quantitative ALP activity at different time points during differentiation course of 15 days. Representative images of ALP staining are shown. **d** In vivo ectopic bone formation by mOBs versus 2 independent OBs-FS cell lines (at 50 PDL, p30). Cells were mixed with HA/TCP powder and implanted subcutaneously in immune-deficient mice for 2 months (n = 6 implants for each cell line). Images of H&E sections of implants show the formation of ectopic bone by implanted cells. Bone histomorphometric analysis of the percentage of total bone area per total area of the implants. Data are mean ± SD of three independent experiments, (*p < 0.05, **p < 0.005, compared to mBOs control). For panel **c**, columns with different letters at each time point indicate significant differences according to Duncan’s multiple range tests at *p* < 0.05

To examine whether, the above BMSCs culture protocol can be applied for establishing long-term culture of other bone related cells, we cultured the primary murine neonatal OBs by using FS protocol in the presence of bFGF (5 ng/mL). As shown in Fig. 6a, OBs-FS displayed long term culture with more than 70 PDL, while OBs cultured with traditional protocol (see M&M) experienced replicative senescent after 10 PDL. In addition, OBs-FS at PDL 50 (p30) showed homogenous cell population with cuboidal shape like structure as compared with primary OBs at PDL 10 (p7) (Fig. 6b). We further examined in vitro and in vivo osteoblast differentiation of OBs-FS. At PDL 50 (p30), OBs-FS retained their in vitro differentiation potential into osteoblast as assessed by increased ALP activity and Alizarin red staining for matrix mineralization (Fig. 6c). In addition, OBs-FS at PDL 50 (p30) showed to form significant high percentage of ectopic bone in vivo upon implantation with HA/TCP powder in immune-deficient mice as compared with pOBs (Fig. 6d).

## Discussion

In this report, we described a fast and reliable protocol for long term culturing of primary BMSCs, that combines frequent-subculture and bFGF. This method affords high yield of BMSCs, while maintaining their in vitro and in vivo stemness. In addition, our protocol proved to be applicable for culturing primary murine OBs with long life span and high in vitro and in vivo osteoblast differentiation potentials. Our simple and fast protocol will allow to culture large numbers of BMSCs/OBs from transgenic mouse models, for mechanistic studies and translational research applications in the bone field.

We and the others have demonstrated BMSCs, as a group of heterogeneous subpopulations that differs in their in vitro and in vivo proliferation and differentiation capacities [19–21]. However, several protocols that succeeded for generating mBMSCs with long life span were mainly dependent on selecting specific subpopulations with high proliferation/differentiation potential [5, 17, 22]. Such selection for particular BMSCs subpopulations might influence the use of BMSCs in translational research aspects, and for identifying osteogenic mechanism in particular transgenic mouse model. In contrast to other protocols, our FS method, at fixed split ratio provides long-term culture of BMSCs with whole population by avoiding the utilization of any depletion step to remove senescent cells or to select for any particular subpopulation.

Our data demonstrated that bFGF supplementation was very efficient to promote proliferation, extend life span of BMSCs and maintain differentiation of BMSCs. In consistent, bFGF supplementation has been used as a mitogenic factor to promote self-renewing and inhibit cellular senescence of stem cells [3, 23, 24]. For example, bFGF was used in a combination with bone-marrow-conditioned medium [25], and hypoxia [26] to expand BMSCs in long term culture and to maintain their stemness. The action of FGF to inhibit cellular senescence and to promote proliferation of mouse BMSC was found to be mediated through the suppression of PI3K/AKT-MDM2 pathway [27] and activation of ERK pathway respectively [26, 28]. In this context, our protocol showed several advantages over others that have used bFGF supplementation, including; 1) It does not need any special requirements like hypoxic condition or selection for specific sub-populations with unique growing potential [26–28]; 2) it results in high yields of immortalized BMSCs with retained in vitro and in vivo stemness. 3) it is fast and straightforward protocol, that proved to be applicable for culturing other bone-related cell types including osteoprogenitors.

Furthermore, our finding that bFGF maintained the in vivo multipotency differentiation of BMSCs to form ectopic bone and bone marrow stroma, suggesting a role for bFGF in enhancing the regenerative repair of BMSCs. In support to this notion, bFGF treatment of hyaluronic acid-based polymer scaffold in combination with BMSCs was reported to promote the healing of large segmental bone defects in a rat model [29]. Furthermore, bFGF was shown to enhance the therapeutic efficacy of transplanted BMSCs for myocardial infarction in pre-clinical animal model [30, 31]. Thus, our protocol could provide high yield of BMSCs with enhanced potential for bone repair. However, further in vivo studies are needed to investigate the therapeutic potential of BMSCs for bone repair in animal model of bone defect.

Several studies have demonstrated long-term culture of plastic-adherent mouse BMSCs [4, 25, 32]. On the other hand, some reports demonstrated that mouse BMSCs might exhibit chromosomal instability and in vivo tumorigenicity, in a mechanism mediated by p53 mutation [33–36]. In this context, we used in vivo ectopic bone formation assay as a gold standard assay for testing the stemness of mBMSCs in vivo as well as to observe any possibility for in vivo tumor formation upon cell transplantation. In contrast, most culture protocols for generating immortalized BMSCs did not examine the in vivo bone regenerative capacity of cultured mBMSCs [4, 17, 25, 32, 37, 38]. Interestingly, our in vivo data demonstrated that both BMSCs-FS and OBs-FS cell lines have the potential to form heterotropic bone in vivo upon transplantation with HA/TCP with no sign of tumor formation. Despite these data is not fully adequate to exclude the possibility of any genetic instability led to transformation in BMSCs or OBs-FS, our in vivo data were significant enough to demonstrate that our straightforward and fast protocol could provide robust BMSCs safe to be used in pre-clinical applications including cell therapy and bone-tissue engineering.

## Conclusion

In summary, our data provide a simple protocol for long term culturing of mouse BMSCs and OBs based on using FS in the presence of low dose of bFGF. This protocol proved to be simple and highly reproducible to yield homogenous populations of BMSCs and OBs with robust differentiation capacity into osteogenic cell lineage in vitro and in vivo at high PD levels. Unlike other reported protocols, the cultured BMSCs and OBs using our protocol showed no sign of tumorigenicity upon in vivo transplantation in mice, suggesting their safe use in translational research applications.

## Methods

### Isolation and cultivation of BMSCs

The ethical approval for ex-vivo and in vivo animal procedures were approved by the Danish Animal Ethical committee. Primary mouse BMSCs (mBMSCs) were isolated from the bone marrow of wild-type 8-weeks-old C57BL/6J mice as described previously [17]. In brief, the femur and tibia were dissected from mice and bone marrow was flushed out with complete isolation media (CIM), which consists of RPMI-1640 (Roswell Park Memorial Institute), supplemented with 10% fetal bovine serum (FBS; GIBCO), 100 U/mL penicillin (Gibco, Thermo Fisher Scientific, Darmstadt, Germany) and 100 μg/mL streptomycin (GIBCO). Cells were washed with PBS, filtered (through 70 μM nylon mesh filter) and cultured in 40 mL CIM in a 175-cm2 flask in 5% CO2 incubator at 37 °C. Non-adherent cells were removed after 24 h, passage 0 (p0) and sub-cultured in 10 ml of fresh CIM in 60-cm2 flask (p1). Cells were passaged every 1 week using 0.25% trypsin/1 mM EDTA.

For our frequent subculture (FS) protocol, BMSCs (p1) at 90% confluence were sub-cultured at split ratio 1:2 in complete culture medium (CCM) consist of Iscove modified Dulbecco medium (IMDM; GIBCO) supplemented with 10% FBS, bFGF (5 ng/mL), 100 U/ml penicillin, 100 μg/ml streptomycin (GIBCO), and 12 μM l-glutamine (GIBCO). BMSCs were continued to be sub-cultured always (at 90% cell confluence) at split ratio 1:2 in CCM supplemented with bFGF (5 ng/mL) for several passages.

Mice OBs were isolated from the calvarias of neonatal pups (3 to 4 days old) as described [39]. Briefly, calvarias were dissected and subjected to sequential collagenase II digestion at 37 °C. Cells (p0) were cultured in complete osteoprogenitor medium (COM) consists of Dulbecco’s modified Eagle’s medium (DMEM; Gibco) with 20% fetal bovine serum (FBS) and 100 mg/mL of streptomycin (Gibco) and 100 U/mL of penicillin (Gibco).

For our FS protocol, OBs (p1) at 90% cell confluence were sub-cultured in COM supplemented with bFGF (5 ng/mL) at split ratio 1:2 in COM supplemented with bFGF (5 ng/mL). OBs were continued to be sub-cultured (when reached 90% cell confluence) at split ratio 1:2 in the same medium for long term.

Mouse stromal cell line ST2 was obtained from Leibniz Institute DSMZ-German Collection of Microorganisms and Cell Cultures (ACC 333, Braunschweig, Germany). Cells were cultured in DMEM supplemented with 10% fetal bovine serum (FBS) and 1% penicillin/streptomycin (P/S) (Gibco Invitrogen, USA).

### Osteoblast differentiation

Cells were cultured at 15,000 cells/cm2 in basal culture medium. At 70% cell confluence, cultured media were changed to osteogenic induction medium (OIM) consists of: α-minimum essential medium (α-MEM; Gibco) containing 10% FBS, 100 U/mL of penicillin, 100 mg/mL of streptomycin, 50 μg/mL of vitamin C (Sigma-Aldrich), 10 nM dexamethasone and 10 mM β-glycerol-phosphate (Sigma-Aldrich). The media were changed every 2–3 days during the culture.

### Adipocyte differentiation

Cells were cultured at 15,000 cells/cm2 in basal culture medium. At 100% cell confluence, cultured media were replaced by adipogenic induction medium (AIM) consists of: DMEM supplemented with 9% horse serum, 250 nM dexamethasone, 450 μM 1-methyl-3-isobutylxanthine (IBMX), 1 μM rosiglitazone (BRL 49653, Cayman Chemical) and 5 μg/mL insulin (Sigma-Aldrich). The media were changed every 2–3 days during the time course of adipocyte differentiation.

### Alkaline phosphatase (ALP) activity

ALP activity was determined following the manual instructions of ALP assay kit (Abcam plc, Cambridge, UK). The color of the reaction was measured at 405 nm. The number of viable cells was determined using the Cell Titer-Blue cell viability assay according to the manufacturer’s instructions (Promega, USA) at OD 579. ALP activity was represented as fold change over control non-induced cells after normalization to the number of viable cells [40].

### Alizarin red staining for mineralized matrix

Cells were induced to osteoblast differentiation for 12 days. Cells were fixed with 70% ice-cold ethanol for 1 h at − 20 °C, and stained with 40 mM Alizarin red S (AR-S; Sigma-Aldrich), pH 4.2 for 10 min at room temperature. Alizarin red stain was eluted using 10% (*w*/*v*) cetylpyridinium chloride solution (Sigma-Aldrich) with shaking for 20 min and the absorbance of the eluted dye was measured at 570 nm.

### Oil Red O staining and quantification

Cells were induced to adipocyte differentiation and then fixed in 4% paraformaldehyde for 10 min at room temperature. Cells were stained with Oil Red O (0.5 g in 60% isopropanol) (Sigma-Aldrich) for 1 h at room temperature to stain the fat droplets. Lipids were quantified by elution of Oil Red O in isopropanol for 10 min at room temperature. The absorbance of the extracted dye was detected at 490 nm. Oil Red O measurements were normalized to cell umber (measured by number of viable cells) and then represented as fold change over control non-induced cells.

### RNA extraction and real-time PCR analysis

Total RNA was extracted from cells using single-step method of TRIzol (Thermo Fisher Scientific) followed by phenol-chloroform extraction procedure. cDNA was synthesized from 1 μg of total RNA using revertAid H minus first strand cDNA synthesis kit (Fermentas). Quantitative real time PCR was performed with Applied Biosystems 7500 Real-Time system using Fast SYBR® Green Master Mix (Applied Biosystems, California, USA) with specific primers (Additional file 1: Table S1). The expression of each target gene was normalized to *β-Actin* and *Hprt* mRNA expression as reference genes, using a comparative CT method [(1/ (2delta-CT) formula, where delta-CT is the difference between CT-target and CT-reference] with Microsoft Excel 2007® as described [41].

### PCR array analysis

Total RNA was extracted from mBMSCs and mBMSCs-FS that induced to osteoblast differentiation for 6 days. Osteogenic RT2 Profiler™ PCR array, containing 84 osteoblast-related genes (Qiagen Nordic, Denmark), was performed for each cDNA sample in triplicates using SYBR® Green quantitative PCR method on Applied Biosystems 7500 real-time PCR system. Data were analyzed after normalization to reference genes according to the manufacturer’s instructions.

### Fluorescence activated cell sorting (FACS)

CD surface markers were profiled by incubating the cells in FACS buffer containing pre-conjugated antibodies (see Additional file 1: Table S2) for 20 min on ice. Cells were washed twice with FACS buffer and the cell acquisition was performed with flow cytometer BD FACS LSRII (BD Biosciences, Albertslund, Denmark). The data were analyzed using Kaluza®1.2 software (Beckman Coulter Inc.).

### In vivo ectopic bone formation assay

Cells were cultured in CIM medium and 5 × 10^5^ cells, mixed with 40 mg hydroxyapatite/ tricalcium phosphate (HA/TCP) ceramic powder (Zimmer Scandinavia Albertslund, Denmark) and implanted subcutaneously in 2-month-old NOD/MrkBomTac-Prkdcscid female mice (Taconic, Ry, Denmark) (*n* = 6 implants/cell line). Implants demineralized in EDTA solution ((25% *W*/*V*), pH = 7.1), paraffin embedded, sectioned, and stained by eosin/hematoxylin. The percentage of total bone area per total implant area was quantified as described previously [18].

### Statistical analysis

All values are expressed as mean ± SD (standard deviation) of at least three independent experiments. Student’s t-test was used for comparison between two groups. Differences were considered statistically significant at *P < 0.05, and **P < 0.005. In some cases, the data were also statistically analyzed using One-way analysis of variance (ANOVA) and differences among the means were determined for significance at *P* ≤ 0.05 using Duncan’s multiple range test (by SPSS, 16.1 Chicago, USA).

## Additional file

Additional file 1: **Table S1.** List of primers used for qRT-PCR. **Table S2.** Full osteogenic gene expression list (total 84 genes) by BMSCs-FS (p25) versus ST2 cells during osteoblast differentiation including all significant/non-significant pathways. (DOCX 20 kb)

## Abbreviations

AIM: Adipogenic induction medium
ALP: Alkaline phosphatase
*aP2*: adipocyte protein 2
*Apm1*: Adiponectin
AR-S: Alizarin red S
bFGF: Basic fibroblast growth factor
BMPs: Bone morphogenetic proteins
BMSCs: Bone marrow derived stromal stem cells
*C/ebpα*: Ccaat-enhancer-binding protein alfa
cAMP: Cyclic adenosine monophosphate
CCM: Complete culture medium
*Dlx5*: Distal-less homeobox 5
FS: Frequent subculture
HPCs: Hematopoietic progenitors
IBMX: Isobutylxanthine
IGF-1: Insulin growth factor 1
IMDM: Iscove modified Dulbecco medium
*Msx2*: Msh homeobox 2
OBs: Primary neonatal calvarial osteoprogenitor cells
*Ocn*: Osteocalcin
*Opn*: Osteopontein
P: Passage
PD: Population doubling
PDGF: Platelet-derived growth factor
*Pparγ2*: Peroxisome proliferator-activated receptor gamma2
RPMI-1640: Roswell Park Memorial Institute
*Runx2*: Runt-related transcription factor 2
SD: Standard deviation
TGFβ: Transforming growth factor beta
VEGF: Vascular endothelial growth factor
Wnt3a: Wnt family protein
