# Supplementary material for: A simple and reliable protocol for long-term culture of murine bone marrow stromal (mesenchymal) stem cells that retained their in vitro and in vivo stemness in long-term culture
Source: Biol Proced Online. 2019 Feb 1;21:3. doi: 10.1186/s12575-019-0091-3 (PMC6357407; doi:10.1186/s12575-019-0091-3)
Supplement: Supplementary file 1 — Table S1. List of primers used for qRT-PCR. Table S2. Full osteogenic gene expression list (total 84 genes) by BMSCs-FS (p25) versus ST2 cells during osteoblast differentiation including all significant/non-significant pathways. (DOCX 20 kb) [file 12575_2019_91_MOESM1_ESM.docx]

**Additional file 1: Table S1**: List of primers used for qRT-PCR

| **Gene symbol** | **Forward primer 5’-3’** | **Reverse Primer 5’-3’** |
| --- | --- | --- |
| ***β-Actin*** | GAT ATC GCT GCG CTG GTC GTC | ACG CAG CTC ATT GTA GAA GGT GTG G |
| ***Hprt*** | TCAGTCAACGGGGGACATAAA | GGGGCTGTACTGCTTAACCAG |
| ***PPAR-γ*** | GGG TCA GCT CTT GTG AAT GG | CTG ATG CAC TGC CTA TGA GC |
| ***C/ebp-α*** | AAG CCA AGA AGT CGG TGG A | CAG TCC ACG GCT CAG CTG TTC |
| ***aP2*** | CAA AAT GTG TGA TGC CTT TGT G | CTC TTC CTT TGG CTC ATG CC |
| ***Apm1*** | GAC GTT ACT ACA ACT GAA GAG C | CAT TCT TTT CCT GAT ACT GGT C |
| ***Runx2*** | AGC AAC AGC AAC AAC AGC AG | GTA ATC TGA CTC TGT CCT TG |
| ***Ocn*** | CAG ACA AGT CCC ACA CAG CA | CTT TAT TTT GGA GCT GCT GT |
| ***Alp*** | GCC CTC TCC AAG ACA TAT A | CCA TGA TCA CGT CGA TAT CC |
| ***Opn*** | GAA ACT CTT CCA AGC AAT TC | GGA CTA GCT TGT CCT TGT GG |
| ***Msx2*** | CCATATACGGCGCATCCTACC | CAACCGGCGTGGCATAGAG |

**Table S2:** Full osteogenic gene expression list (total 84 genes) by BMSCs-FS (p25) versus ST2 cells during osteoblast differentiation including all significant/non-significant pathways.

| **Gene** | **Gene symbol** | **Fold** |
| --- | --- | --- |
| Activin A receptor, type 1 | *Acvr1* | 1.2 |
| Alpha-2-HS-glycoprotein | *Ahsg* | 1.1 |
| Alkaline phosphatase, liver/bone/kidney | *Alpl* | **7.9** |
| Annexin A5 | *Anxa5* | 0.8 |
| Bone gamma carboxyglutamate protein | *Bglap* | **2.1** |
| Biglycan | *Bgn* | **2** |
| Bone morphogenetic protein 1 | *Bmp1* | 1.3 |
| Bone morphogenetic protein 2 | *Bmp2* | **74** |
| Bone morphogenetic protein 3 | *Bmp3* | **25.3** |
| Bone morphogenetic protein 4 | *Bmp4* | **2** |
| Bone morphogenetic protein 5 | *Bmp5* | **2** |
| Bone morphogenetic protein 6 | *Bmp6* | **17.7** |
| Bone morphogenetic protein 7 | *Bmp7* | 1.2 |
| Bone morphogenetic protein receptor, type 1A | *Bmpr1a* | 1.7 |
| Bone morphogenetic protein receptor, type 1B | *Bmpr1b* | **3.8** |
| Bone morphogenic protein receptor, type II (serine/threonine kinase) | *Bmpr2* | **1.2** |
| CD36 antigen | *Cd36* | 0.75 |
| Cadherin 11 | *Cdh11* | 0.84 |
| Chordin | *Chrd* | 0.62 |
| Collagen, type X, alpha 1 | *Col10a1* | 1.42 |
| Collagen, type XIV, alpha 1 | *Col14a1* | 1.2 |
| Collagen, type I, alpha 1 | *Col1a1* | **7.5** |
| Collagen, type I, alpha 2 | *Col1a2* | **4.2** |
| Collagen, type II, alpha 1 | *Col2a1* | **7.5** |
| Collagen, type III, alpha 1 | *Col3a1* | **2.2** |
| Collagen, type IV, alpha 1 | *Col4a1* | **2.2** |
| Collagen, type V, alpha 1 | *Col5a1* | **2.5** |
| Cartilage oligomeric matrix protein | *Comp* | 0.94 |
| Colony stimulating factor 1 (macrophage) | *Csf1* | **3** |
| Colony stimulating factor 2 (granulocyte-macrophage) | *Csf2* | 0.98 |
| Colony stimulating factor 3 (granulocyte) | *Csf3* | 0.88 |
| Cathepsin K | *Ctsk* | 1.1 |
| Distal-less homeobox 5 | *Dlx5* | 0.75 |
| Epidermal growth factor | *Egf* | 0.95 |
| Fibroblast growth factor 1 | *Fgf1* | **2.5** |
| Fibroblast growth factor 2 | *Fgf2* | **2.7** |
| Fibroblast growth factor receptor 1 | *Fgfr1* | **4.4** |
| Fibroblast growth factor receptor 2 | *Fgfr2* | **2.3** |
| FMS-like tyrosine kinase 1 | *Flt1* | 1.2 |
| Fibronectin 1 | *Fn1* | 0.86 |
| Growth differentiation factor 10 | *Gdf10* | **3.7** |
| GLI-Kruppel family member GLI1 | *Gli1* | 1.5 |
| Intercellular adhesion molecule 1 | *Icam1* | 0.68 |
| Insulin-like growth factor 1 | *Igf1* | **2** |
| Insulin-like growth factor I receptor | *Igf1r* | **2.2** |
| Indian hedgehog | *Ihh* | 1.1 |
| Integrin alpha 2 | *Itga2* | **10** |
| Integrin alpha 2b | *Itga2b* | 1.5 |
| Integrin alpha 3 | *Itga3* | 1.1 |
| Integrin alpha M | *Itgam* | 1.35 |
| Integrin alpha V | *Itgav* | 1.28 |
| Integrin beta 1 (fibronectin receptor beta) | *Itgb1* | 0.93 |
| Matrix metallopeptidase 10 | *Mmp10* | 1.2 |
| Matrix metallopeptidase 2 | *Mmp2* | **17** |
| Matrix metallopeptidase 8 | *Mmp8* | **12** |
| Matrix metallopeptidase 9 | *Mmp9* | **23.7** |
| Nuclear factor of kappa light polypeptide gene enhancer in B-cells 1, p105 | *Nfkb1* | 1.2 |
| Noggin | *Nog* | 0.85 |
| Platelet derived growth factor, alpha | *Pdgfa* | **3.2** |
| Phosphate regulating gene with homologies to endopeptidases on the X chromosome (hypophosphatemia, vitamin D resistant rickets) | *Phex* | 0.95 |
| Runt related transcription factor 2 | *Runx2* | **4.5** |
| Serine (or cysteine) peptidase inhibitor, clade H, member 1 | *Serpinh1* | 1 |
| MAD homolog 1 (Drosophila) | *Smad1* | 1.1 |
| MAD homolog 2 (Drosophila) | *Smad2* | 1.6 |
| MAD homolog 3 (Drosophila) | *Smad3* | 1.3 |
| MAD homolog 4 (Drosophila) | *Smad4* | 0.64 |
| MAD homolog 5 (Drosophila) | *Smad5* | 0.82 |
| Sclerostin | *Sost* | 1.2 |
| SRY-box containing gene 9 | *Sox9* | 1.1 |
| Sp7 transcription factor 7 | *Sp7* | **6.7** |
| Secreted phosphoprotein 1 | *Spp1* | 0.95 |
| Transforming growth factor, beta 1 | *Tgfb1* | 1 |
| Transforming growth factor, beta 2 | *Tgfb2* | 0.93 |
| Transforming growth factor, beta 3 | *Tgfb3* | 0.94 |
| Transforming growth factor, beta receptor I | *Tgfbr1* | 1.1 |
| Transforming growth factor, beta receptor II | *Tgfbr2* | 1.1 |
| Transforming growth factor, beta receptor III | *Tgfbr3* | 1.4 |
| Tumor necrosis factor | *Tnf* | 1.24 |
| Tumor necrosis factor (ligand) superfamily, member 11 | *Tnfsf11* | 1.1 |
| Twist homolog 1 (Drosophila) | *Twist1* | 0.82 |
| Vascular cell adhesion molecule 1 | *Vcam1* | 0.84 |
| Vitamin D receptor | *Vdr* | 0.97 |
| Vascular endothelial growth factor A | *Vegfa* | 0.95 |
| Vascular endothelial growth factor B | *Vegfb* | 1.1 |
| Heat shock protein 90 alpha (cytosolic), class B member 1 | *Hsp90ab1* | 1 |

Cells were induced into osteoblast differentiation as described in the M&M. Mouse osteogenesis RT² Profiler™ PCR array with 84 osteogenic genes was performed using the SYBR® Green quantitative PCR method. Each target gene was normalized to a group of reference genes as described by manufacture instructions and data are represented as fold change over ST2 cells. Up-regulated genes (≥ 2 fold) by BMSCs-FS are in bold as fold change over ST2 cells.
